# Supplementary material for: The metabolic enzyme hexokinase 2 localizes to the nucleus in AML and normal haematopoietic stem and progenitor cells to maintain stemness
Source: Nat Cell Biol. 2022 Jun 6;24(6):872–84. doi: 10.1038/s41556-022-00925-9 (PMC9203277; doi:10.1038/s41556-022-00925-9)
Supplement: Supplementary file 1 — Reporting Summary [file 41556_2022_925_MOESM1_ESM.pdf]

nature research

Corresponding author(s): Aaron Schimmer

Last updated by author(s): Mar 11, 2022

Reporting Summary

Nature Research wishes to improve the reproducibility of the work that we publish. This form provides structure for consistency and transparency in reporting. For further information on Nature Research policies, see our Editorial Policies and the Editorial Policy Checklist.

Statistics

For all statistical analyses, confirm that the following items are present in the figure legend, table legend, main text, or Methods section.

n/a	Confirmed	
☐	☒	The exact sample size (*n*) for each experimental group/condition, given as a discrete number and unit of measurement
☐	☒	A statement on whether measurements were taken from distinct samples or whether the same sample was measured repeatedly
☐	☒	The statistical test(s) used AND whether they are one- or two-sided<br>*Only common tests should be described solely by name; describe more complex techniques in the Methods section.*
☒	☐	A description of all covariates tested
☒	☐	A description of any assumptions or corrections, such as tests of normality and adjustment for multiple comparisons
☐	☒	A full description of the statistical parameters including central tendency (e.g. means) or other basic estimates (e.g. regression coefficient) AND variation (e.g. standard deviation) or associated estimates of uncertainty (e.g. confidence intervals)
☐	☒	For null hypothesis testing, the test statistic (e.g. *F*, *t*, *r*) with confidence intervals, effect sizes, degrees of freedom and *P* value noted<br>*Give P values as exact values whenever suitable.*
☒	☐	For Bayesian analysis, information on the choice of priors and Markov chain Monte Carlo settings
☒	☐	For hierarchical and complex designs, identification of the appropriate level for tests and full reporting of outcomes
☐	☒	Estimates of effect sizes (e.g. Cohen's *d*, Pearson's *r*), indicating how they were calculated

Our web collection on statistics for biologists contains articles on many of the points above.

Software and code

Policy information about availability of computer code

Data collection	Confocal Imaging: Leica X Software, Flow cytometry: BD FACS Diva Software, RPPA: Microvigene Software v3.0, MTS Assay: Softmax Pro v7.0.3.
Data analysis	Statistical Analysis: Graph Pad Prism, Flow cytometry: Flow Jo V7.7 & V10.7;<br>Confocal imaging: Image J 2.3.0, LAS AF, Metamorph v7.8;<br>Comet Assay: Open Comet 1.3.1,<br>Densitometry: Image Lab 5.2.1, GREAT_4.0.4 (great.Stanford.edu)<br>RNA Seq:Reads were aligned against hg38 using Tophat v. 2.0.11. Read counts per gene were ob-tained through htseq-count v.0.6.1p2 in the mode ‘‘intersection nonempty.’’ EdgeR R package v.3.16.5 was used to normalize the data using the TMM (trimmed mean of M values) method and to estimate differential expression. R package GSVA_1.30.0 was used to analyze LSC+/LSC- signatures. Differential expression between LSC+ and LSC- fractions was calculated the limma R package 3.28.21.<br>BioID: RAW files were converted to the .mzML format using ProteoWizard (v3.0.10800) (43) and then searched using X! Tandem [X! TANDEM Jackhammer TPP (v2013.06.15.1)] 59 and Comet (v2014.02 rev.2) 60 against the human Human RefSeq v45 database (containing 36113 entries). Data were filtered through the trans-proteomic pipeline (TPP) (v4.7 POLAR VOR-TEX rev 1) .<br>ATAC seq: ATAC samples were preprocessed according to the ENCODE ATAC-seq pipeline. Single-end reads were aligned to the hg38 genome using Bowtie2 64, reads with MAPQ scores < 30 were filtered out with Samtools 65, duplicates were removed using Sambamba 66 and TN5 tagAlign shifted files were created. MACS2 67 was used to call peaks. Peak counts and sizes for each replicate were calculated using a custom Python script, and Jaccard indices for similarities between called peaks was calculated using BEDTools 68. Differentially accessible regions were calculated using the DiffBind and EdgeR 69 packages in R. Differentially accessible regions (DAR) were mapped to genes using the annotatePeak function of the R package ChIPseeker_1.22.1. Feature Distribution were plotted using the function plotAnnoBar. DARs were subjected to pathway analysis using the GREAT tool version 4.0.4. Pathways enriched at FDR 0.05 belonging to the category of GO Biological Processes (BP) were visualized as a network using Cytoscape 3.8.1 and EnrichmentMap 3.3.1and AutoAnnotate 1.3.3.<br>ChIP seq: Prior to analysis, read adapters were removed using Trim_Galore v. 0.4.0. Reads were aligned against hg38 (UCSC version) using Bowtie2 v2.3.2. Alignment reads were deduplicated to remove duplicate reads and keep unique reads us-ing picard v. 1.9.1. Broad peaks were

identified from the alignment files using MACS2 v.2.1.1. Peaks were annotated with all the potential genomic features based on hg38 GENCODE v24 gene assembly which was downloaded from UCSC database. MACS2 called peaks at FRD 0.01 for individual samples and pooled samples were sub-jected to pathway analysis using the GREAT tool version 4.0.4. Overlapping peaks for the 2 replicates of MAX (ENCFF793GVV.bed) using the function findOverlappingPeaks from the ChIPpeakAnno_3.20.1 R package. The findMotifsGenome algorithm from HOMER v4.7 was used to identify known enriched motifs in genomic regions. consensus sequences of motifs were aligned using MAFFT (https://mafft.cbrc.jp/alignment/software/). TFmotifView (http://bardet.u-strasbg.fr/) was used to calculate the significance of motifs.

For manuscripts utilizing custom algorithms or software that are central to the research but not yet described in published literature, software must be made available to editors and reviewers. We strongly encourage code deposition in a community repository (e.g. GitHub). See the Nature Research guidelines for submitting code & software for further information.

Data

Policy information about availability of data

All manuscripts must include a data availability statement. This statement should provide the following information, where applicable:

- Accession codes, unique identifiers, or web links for publicly available datasets
- A list of figures that have associated raw data
- A description of any restrictions on data availability

NCBI Accession number: NM_000189.5
BioGRID: https://thebiogrid.org/109346/summary/homo-sapiens/hk2.html
Omnibus dataset GSE24759
Gene Expression Omnibus dataset GSE76008
RNA seq-GSE176103
ATAC seq- GSE176071
ChIP seq- GSE176072

Field-specific reporting

Please select the one below that is the best fit for your research. If you are not sure, read the appropriate sections before making your selection.

☒ Life sciences ☐ Behavioural & social sciences ☐ Ecological, evolutionary & environmental sciences

For a reference copy of the document with all sections, see nature.com/documents/nr-reporting-summary-flat.pdf

Life sciences study design

All studies must disclose on these points even when the disclosure is negative.

Sample size	No statistical methods were used to predetermine sample sizes, but our sample sizes are similar to those reported in previous publications.
Data exclusions	Data were not excluded
Replication	Data were replicated in at least 3 biologic replicates and technical replicates. All experiments were reproducible. Key experiments were reproduced by different individuals
Randomization	For the in vivo experiments, the mice were randomly assigned to Control vs overexpression/knockdown models prior to intervention. The assignment and treatment of the mice was performed by an individual who was not involved in the analysis of the data from the experiment. For in vitro experiments, randomization was not applied to allocate samples into experimental groups.
Blinding	In vivo experiments were not blinded, however, experiments and analysis were performed independently. For in vitro cell based experiments, the cell type and treatment condition were known because the experiments required the investigators to group the data between control and testing conditions to quantify differences. However, key experiments were reproduced independently by different individuals.

Reporting for specific materials, systems and methods

We require information from authors about some types of materials, experimental systems and methods used in many studies. Here, indicate whether each material, system or method listed is relevant to your study. If you are not sure if a list item applies to your research, read the appropriate section before selecting a response.

Materials & experimental systems

n/a	Involved in the study
☐	☒ Antibodies
☐	☒ Eukaryotic cell lines
☒	☐ Palaeontology and archaeology
☐	☒ Animals and other organisms
☐	☒ Human research participants
☒	☐ Clinical data
☒	☐ Dual use research of concern

Methods

n/a	Involved in the study
☐	☒ ChIP-seq
☐	☒ Flow cytometry
☒	☐ MRI-based neuroimaging

Antibodies

Antibodies used

Hexokinase II (C64G5) Rabbit mAb Cell signalling Cat #2867 WB 1:1000, IF 1:200
Mouse monoclonal anti- beta-Actin (AC-15) Santa Cruz Biotechnology Cat# sc-69879 WB 1:10000
Rabbit polyclonal anti-beta Tubulin (H-235) Santa Cruz Biotechnology Cat# sc-9104 WB 1:5000
Histone H3 (D1H2) XP® Rabbit mAb Cell signalling Cat #4499 WB 1:5000
Mn SOD polyclonal antibody Enzo Cat#ADI-SOD-110-F WB 1:3000
Monoclonal ANTI-FLAG® M2 antibody produced in mouse Sigma Cat#F3165 WB 1:1000
53BP1 Antibody Novus Biologics Cat#NB100-304 IF 1:600
Anti-HK II Antibody (B-8) Santa Cruz Biotechnology Cat#sc-374091 WB 1:1000
Anti-Aldolase A Antibody (C-10) Santa Cruz Biotechnology Cat#sc-390733 WB 1: 500
ACO2 Antibody Cell signalling Cat#6922 WB 1: 1000
Rabbit monoclonal anti-citrate synthase Abcam Cat# ab129095 WB 1: 1000
Enolase-1 Antibody Cell signalling Cat#3810 WB 1: 1000
GAPDH (14C10) Rabbit mAb Cell signalling Cat#2118 WB 1: 1000
PFKP Antibody Cell signalling Cat#5412 WB 1: 500
IPO5 Antibody Thermo PA5-30076 WB 1: 1000
PHLPP1 Antibody Proteintech 22789-1-AP WB 1: 1000
PKM2 Antibody Abcam Cat#3198 WB 1: 1000
GPI Antibody Thermo Scientific Cat#PA5-29665 WB 1: 1000
Hexokinase I (C35C4) Rabbit mAb Cell signalling Cat#2024 WB 1: 1000
Anti-Hexokinase Type III/HK3 antibody Abcam Cat#ab91097 WB 1: 1000
Anti-Rad51 antibody Abcam Cat#ab63801 IF 1:600
Anti-phospho-Histone H2A.X (Ser139) Antibody, clone JBW301 EMD Millipore Cat#05-636 IF 1:300
Anti-SDHA antibody [2E3GC12FB2AE2] Abcam Cat#ab14715 WB 1: 1000
Anti-SDHB antibody [EPR13042(B)] Abcam Cat#ab178423 WB 1: 1000
Mouse Anti-Tom20 Clone 29 BD Biosciences Cat#612278 IF 1: 400
Mouse anti-human CD45 BB515 (HI30) BD Biosciences Cat#555413 Flow 1:100
Mouse anti-human CD3 PE-Cy7 (SP34-2) BD Biosciences Cat#557749 Flow 1:100
Mouse anti-human CD19 PE (HIB19) BD Biosciences Cat#555413 Flow 1:100
BB515 Rat Anti-Mouse Ly-6A/E BD Biosciences Cat# 565397 Flow 1:100
Pe/Cy7 Rat anti-mouse CD117 (c-Kit) BioLegend Cat# 105814 Flow 1:100
PE Rat anti-mouse CD150 (SLAM) BioLegend Cat# 115904 Flow 1:100
PE/Cy5 Anti-Mo-LY-6A/E (Sca-1) Invitrogen Cat#15-5981-82 Flow 1:200
APC Hamster anti-mouse CD48 ThermorFisher Scientific Cat# 17-0481-82 Flow 1:50
APC/Cy7 Rat anti-mouse CD16/32 BioLegend Cat# 101328 Flow 1:200
eFluor 450 Rat anti-mouse CD34 ThermoreFisher Scientific Cat# 48-0341-82 Flow 1:200
APC Mouse anti-human CD11b BD Biosciences Cat# 340937 Flow 1:200
APC Mouse anti-human CD14 BD Biosciences Cat# 555399 Flow 1:100
CD34 BD Biosciences Cat# 348053 Flow 1:200
CD38 BD Biosciences Cat# 342371 Flow 1:100
FITC Mouse Anti-Human CD45RA BD Biosciences Cat# 555488 Flow 1:25
APC Mouse Anti-Human CD90 BD Biosciences Cat#561971 Flow 1:50
PE-Cy™7 Mouse Anti-Human CD38 Clone HB7 BD Biosciences Cat#335790 Flow 1:200
V450 Mouse Anti-Human CD7 Clone M-T701 BD Biosciences Cat#642916 Flow 1:50
V500 Mouse Anti-Human CD45 Clone HI30 BD Biosciences Cat#560777 Flow 1:200
APC/Cyanine7 anti-human CD34 Clone 581 BioLegend Cat#343513 Flow 1:100
CD34 Monoclonal Antibody (4H11), PerCP-eFluor 710 eBioscience Cat#46-0349-42 Flow 1:100
CD33-PE-Cy5 anti-human Beckman Coulter Cat#PNIM2647U Flow 1:100
CD3 FITC Clone SK7 BD Biosciences Cat#349201 Flow 1:100
CD19 (Leu™-12) PE Clone 4G7 BD Biosciences Cat#349209 Flow 1:200
Alexa Fluor®647 Mouse Anti-Human CD56 Clone B159 BD Biosciences Cat#557711 Flow 1:100
Qdot™ 605 Streptavidin Conjugate Invitrogen Q10101MP Flow 1:200
Biotin anti-human CD135 (Flt-3/Flk-2) BioLegend Cat#313312 Flow 1:10
anti-CD10-Alexa-700 BD Biosciences 624040 Flow 1:10
Pacific Blue™ anti-mouse CD45.1 Antibody Biolegend 110721 Flow 1:100
APC/Cyanine7 anti-mouse CD45.2 Antibody Biolegend 109823 Flow 1:100
MAX (Santa-Cruz, sc-197,1:200), SPIN1 (Cell signaling, 89139, ,1:100), CTR9 (Cell sig-naling, 12619, ,1:100), IWS1 (Cell signaling, 5681, ,1:100)

Validation	Antibodies were validated as per manufactures instructions. In addition, antibodies were validated by knocking down the target with shRNA, demonstrating a reduction in the band by immunoblotting.

Eukaryotic cell lines

Policy information about cell lines

Cell line source(s)	OCI AML2 - Dr. Mark D. Minden, NB4 (ACC207) - DSMZ , K562 – ATCC , U937 - ATCC, TEX – Dr. John Dick, OCI-AML 8227 - Dr. John Dick, 130578 – Dr. Steven Chan, HL60 - ATCC, HEK293 - ATCC, KBM3- Dr. Michael Andreeff, KG1a - ATCC, ML2 -DSMZ, MOLM13 -Dr. Michael Andreeff, MOLM14 -Dr. Michael Andreeff, MV411 -ATCC, OCI-AML3 - DSMZ, OCI-AML5 - DSMZ, SKM1 - Dr. Garcia-Manero Guillermo, THP1 - ATCC, CCRF-CEM - Dr. Yiling Lu, Jukart - ATCC, MOLT4 - Dr. Michael Andreeff, TALL1 - Dr. Michael Andreeff
Authentication	Short Tandem Repeat (STR) Genotyping
Mycoplasma contamination	All cell lines tested negative for mycoplasma
Commonly misidentified lines (See ICLAC register)	No commonly misinterpreted cell lines were used in the study.

Animals and other organisms

Policy information about studies involving animals; ARRIVE guidelines recommended for reporting animal research

Laboratory animals	NOD.Cg-Prkdcscid Il2rgtm1Wjl Tg(CMVIL3,CSF2,KITLG)1Eav/MloySzJ (NOD-SCID-GF) 6-12 week old male or female mice(1:1 ratio) were used for the engraftment studies. 5 week old male SCID mice were used in the tumor progression analysis. (AUP): # 1251.38 (NOD-SCID-GF and SCID mice). 5-10 week old male or female mice Vav NLS HK2 Mouse C57BL6 (CD45.2+) & 5 week old male B6.SJL-Ptprca Pepcb/BoyJ (CD45.1+) were used for the hematopoietic stem progenitor analysis and competitive repopulation assays. (AUP#2244.16) for B6 mice. The mice were housed in micro isolator cages with temperature-controlled conditions under a 12-hour light/dark cycle with access to drinking water and food. All animal studies were performed in accordance with the Ontario Cancer Institute Animal Use Protocol.
Wild animals	Not involved
Field-collected samples	Not involved
Ethics oversight	University Health Network ethical review committee & Ontario Cancer Institute Animal Use Protocol

Note that full information on the approval of the study protocol must also be provided in the manuscript.

Human research participants

Policy information about studies involving human research participants

Population characteristics	This has been included in the Extended Data Table 2.
Recruitment	Primary human AML samples from peripheral blood or the bone marrow of male or female AML patients, were collected after obtaining informed consent.
Ethics oversight	University Health Network (REB # 01-0573) and MD Anderson Cancer Center Institutional Review Board reviewed and approved the collection protocol and the research usage protocol.

Note that full information on the approval of the study protocol must also be provided in the manuscript.

ChIP-seq

Data deposition

☒ Confirm that both raw and final processed data have been deposited in a public database such as GEO.

☒ Confirm that you have deposited or provided access to graph files (e.g. BED files) for the called peaks.

Data access links *May remain private before publication.*	Data have been deposited to Gene EXpression Omni-bus Database , RNA seq - GSE176103, ATAC seq - GSE176071 & ChIP seq - GSE176072.
Files in database submission	RNA Seq<br>PROCESSED DATA FILES<br>8227_GFP_2.tsv<br>8227_GFP_3.tsv<br>8227_SH1_1.tsv<br>8227_SH1_2.tsv<br>8227_SH1_3.tsv

RAW FILES
8227_GFP_2_S4_L002_R1_001.fastq.gz
8227_GFP_2_S4_L001_R2_001.fastq.gz
8227_GFP_3_S7_L001_R1_001.fastq.gz
8227_GFP_3_S7_L001_R2_001.fastq.gz
8227_SH1_1_S2_L001_R1_001.fastq.gz
8227_SH1_1_S2_L001_R2_001.fastq.gz
8227_SH1_2_S5_L001_R1_001.fastq.gz
8227_SH1_2_S5_L001_R2_001.fastq.gz
8227_SH1_3_S8_L001_R1_001.fastq.gz
8227_SH1_3_S8_L001_R2_001.fastq.gz
PAIRED-END EXPERIMENTS
8227_GFP_2_S4_L002_R1_001.fastq.gz
8227_GFP_3_S7_L001_R1_001.fastq.gz
8227_SH1_1_S2_L001_R1_001.fastq.gz
8227_SH1_2_S5_L001_R1_001.fastq.gz
8227_SH1_3_S8_L001_R1_001.fastq.gz

ATAC Seq:
PROCESSED DATA FILES
EFa_1_S18_L001_R1_001.trim.merged.nodup.no_chrM_MT.tn5.pval0.01.300K.bfilt.narrowPeak.gz
EFa_2_S22_L001_R1_001.trim.merged.nodup.no_chrM_MT.tn5.pval0.01.300K.bfilt.narrowPeak.gz
Efa_3_S28_L001_R1_001.trim.merged.nodup.no_chrM_MT.tn5.pval0.01.300K.bfilt.narrowPeak.gz
PAA1_S17_L001_R1_001.trim.merged.nodup.no_chrM_MT.tn5.pval0.01.300K.bfilt.narrowPeak.gz
PAA2_S21_L001_R1_001.trim.merged.nodup.no_chrM_MT.tn5.pval0.01.300K.bfilt.narrowPeak.gz
PAA2_S21_L001_R1_001.trim.merged.nodup.no_chrM_MT.tn5.pval0.01.300K.bfilt.narrowPeak.gz
OM_GFP1_S20_L001_R1_001.trim.merged.nodup.no_chrM_MT.tn5.pval0.01.300K.bfilt.narrowPeak.gz
OM_GFP2_S24_L001_R1_001.trim.merged.nodup.no_chrM_MT.tn5.pval0.01.300K.bfilt.narrowPeak.gz
OM_GFP3_S26_L001_R1_001.trim.merged.nodup.no_chrM_MT.tn5.pval0.01.300K.bfilt.narrowPeak.gz
OM_SH1_1_S19_L001_R1_001.trim.merged.nodup.no_chrM_MT.tn5.pval0.01.300K.bfilt.narrowPeak.gz
OM_SH1_2_S23_L001_R1_001.trim.merged.nodup.no_chrM_MT.tn5.pval0.01.300K.bfilt.narrowPeak.gz
OM_SH1_3_S25_L001_R1_001.trim.merged.nodup.no_chrM_MT.tn5.pval0.01.300K.bfilt.narrowPeak.gz
RAW FILES
EFa_1_S18_L001_R1_001.fastq.gz
EFa_2_S22_L001_R1_001.fastq.gz
Efa_3_S28_L001_R1_001.fastq.gz
PAA1_S17_L001_R1_001.fastq.gz
PAA2_S21_L001_R1_001.fastq.gz
PAA3_S27_L001_R1_001.fastq.gz
OM_GFP1_S20_L001_R1_001.fastq.gz
OM_GFP2_S24_L001_R1_001.fastq.gz
OM_GFP3_S26_L001_R1_001.fastq.gz
OM_SH1_1_S19_L001_R1_001.fastq.gz
OM_SH1_2_S23_L001_R1_001.fastq.gz
OM_SH1_3_S25_L001_R1_001.fastq.gz
EFa_1_S18_L001_R2_001.fastq.gz
EFa_2_S22_L001_R2_001.fastq.gz
Efa_3_S28_L001_R2_001.fastq.gz
PAA1_S17_L001_R2_001.fastq.gz
PAA2_S21_L001_R2_001.fastq.gz
PAA3_S27_L001_R2_001.fastq.gz
OM_GFP1_S20_L001_R2_001.fastq.gz
OM_GFP2_S24_L001_R2_001.fastq.gz
OM_GFP3_S26_L001_R2_001.fastq.gz
OM_SH1_1_S19_L001_R2_001.fastq.gz
OM_SH1_2_S23_L001_R2_001.fastq.gz
OM_SH1_3_S25_L001_R2_001.fastq.gz

ChIP Seq:
PROCESSED DATA FILES
EV1_S1_L002_R1_001.nodup.pval0.01.500K.bfilt.narrowPeak.gz
EV2_S2_L002_R1_001.nodup.pval0.01.500K.bfilt.narrowPeak.gz
EV3_S3_L002_R1_001.nodup.pval0.01.500K.bfilt.narrowPeak.gz
PAA1_S4_L002_R1_001.nodup.pval0.01.500K.bfilt.narrowPeak.gz
PAA2_S5_L002_R1_001.nodup.pval0.01.500K.bfilt.narrowPeak.gz
PAA3_S6_L002_R1_001.nodup.pval0.01.500K.bfilt.narrowPeak.gz
NES1_S7_L002_R1_001.nodup.pval0.01.500K.bfilt.narrowPeak.gz
NES2_S8_L002_R1_001.nodup.pval0.01.500K.bfilt.narrowPeak.gz
NES3_S9_L002_R1_001.nodup.pval0.01.500K.bfilt.narrowPeak.gz
RAW FILES
EV1_S1_L002_R1_001.fastq.gz
EV1_S1_L002_R2_001.fastq.gz
EV2_S2_L002_R1_001.fastq.gz
EV2_S2_L002_R2_001.fastq.gz
EV3_S3_L002_R1_001.fastq.gz
EV3_S3_L002_R2_001.fastq.gz

PAA1_S4_L002_R1_001.fastq.gz
PAA1_S4_L002_R2_001.fastq.gz
PAA2_S5_L002_R1_001.fastq.gz
PAA2_S5_L002_R2_001.fastq.gz
PAA3_S6_L002_R1_001.fastq.gz
PAA3_S6_L002_R2_001.fastq.gz
NES1_S7_L002_R1_001.fastq.gz
NES1_S7_L002_R2_001.fastq.gz
NES2_S8_L002_R1_001.fastq.gz
NES2_S8_L002_R2_001.fastq.gz
NES3_S9_L002_R1_001.fastq.gz
NES3_S9_L002_R2_001.fastq.gz
MD5 (EV1_S1_L002_R1_001.fastq.gz) = 858e3c60355a007941cdfae01c4e6dd5
MD5 (EV1_S1_L002_R2_001.fastq.gz) = 9aa16e7bdebc1b6c5726b5a661fae0d2
MD5 (EV2_S2_L002_R1_001.fastq.gz) = c31cde4e4432927c898db8552a72b933
MD5 (EV2_S2_L002_R2_001.fastq.gz) = afc751195418de45ee5693c98eee7503
MD5 (EV3_S3_L002_R1_001.fastq.gz) = f721682b3625a639b56d781578021486
MD5 (EV3_S3_L002_R2_001.fastq.gz) = 968e879d179ee484bb92838300c7b7b7
MD5 (NES1_S7_L002_R1_001.fastq.gz) = 8af893be7084d8c62b4385ca70ddc7c3
MD5 (NES1_S7_L002_R2_001.fastq.gz) = 89be07d16e0cbce3dae91079e256bb30
MD5 (NES2_S8_L002_R1_001.fastq.gz) = 4e26266ea133b039b32334e70acfe816
MD5 (NES2_S8_L002_R2_001.fastq.gz) = 7a1404ff4ab3ac57c355037829fc4127
MD5 (NES3_S9_L002_R1_001.fastq.gz) = 7dac594817d0948ef66dc8fe203df874
MD5 (NES3_S9_L002_R2_001.fastq.gz) = 8aad1f3bb01c865e2b3e7a582dac5f0e
MD5 (PAA1_S4_L002_R1_001.fastq.gz) = d27f6f772303bbf7f1a8d444846316af
MD5 (PAA1_S4_L002_R2_001.fastq.gz) = 0b6d7e99f5b1860629d8d0cfdba0c001
MD5 (PAA2_S5_L002_R1_001.fastq.gz) = 25f1e3b0119eb4e7bdebe1ef2883fc06
MD5 (PAA2_S5_L002_R2_001.fastq.gz) = 1171d9dd8867e4eb13acde33c62d356e
MD5 (PAA3_S6_L002_R1_001.fastq.gz) = e48999515a6c319f820744810da8a53b
MD5 (PAA3_S6_L002_R2_001.fastq.gz) = 83098efad1946c696748a17844863c1d
MD5 (EV1_S1_L002_R1_001.nodup.pval0.01.500K.bfilt.narrowPeak.gz) = 878140f7492a73495ebce5e86f8b52b3
MD5 (EV2_S2_L002_R1_001.nodup.pval0.01.500K.bfilt.narrowPeak.gz) = 720ed3c41f86486b27e667608e3335ba
MD5 (EV3_S3_L002_R1_001.nodup.pval0.01.500K.bfilt.narrowPeak.gz) = c4e936481bbd1da6097042d8d22b5981
MD5 (NES1_S7_L002_R1_001.nodup.pval0.01.500K.bfilt.narrowPeak.gz) = fad39929407ad1803b26847af57a35df
MD5 (NES2_S8_L002_R1_001.nodup.pval0.01.500K.bfilt.narrowPeak.gz) = aea2fb7e6bc5509d62edd40c50dacf9d
MD5 (NES3_S9_L002_R1_001.nodup.pval0.01.500K.bfilt.narrowPeak.gz) = 9d77cfc1f24e57a76dc6e19b9fdfb5f9
MD5 (PAA1_S4_L002_R1_001.nodup.pval0.01.500K.bfilt.narrowPeak.gz) = 8d54ae9ec993498e43c57607d8fd571c
MD5 (PAA2_S5_L002_R1_001.nodup.pval0.01.500K.bfilt.narrowPeak.gz) = 357dfae969bbe0bd36423f07857c22f9
MD5 (PAA3_S6_L002_R1_001.nodup.pval0.01.500K.bfilt.narrowPeak.gz) = 91f188340369355731bb8a8d93a94a51

Genome browser session
(e.g. UCSC)

GEO

Methodology

Replicates

3 Biological replicates

Sequencing depth

		rep1	rep2	rep3
EV	Total Reads	89178502	67951346	68028648
	Mapped Reads	85343440	66582031	66131862
NLS1 HK2				
	Total Reads	60189652	77018382	75795786
	Mapped Reads	56367586	74916850	72362159
NLS2 HK2				
	Total Reads	63210334	63827492	70883392
	Mapped Reads	61136782	62631788	69070112

Antibodies

Anti-HK II Antibody (B-8), Santa Cruz Biotechnology Cat#sc-374091

Peak calling parameters

Peak-calling on replicates and pooled data was carried out using MACS2 (v2.1.0). A p-value threshold of 0.01 was used for MACS2. Output files from MACS2 peak-calling include narrowpeak files, fold-enrichment and -log10(p-value) bigwig files. Once all bed files were converted to bigbed format, naïve overlap thresholding for the MACS2 peak calls was run.

Data quality

The initial quality control metrics for the ChIP-seq data were obtained using the tool FastQC (v0.11.5). All samples were processed using the official ChIP-seq pipeline specification of the Encyclopedia of DNA Elements (ENCODE) consortium, with QC report generated confirming quality metrics including alignment/peak statistics .The single-end BAM files were converted to tagAlign format (BED 3+3 format) and cross-correlation QC scores were calculated using phantompeakqualtools (v1.2).

Software

Read adapters were removed using Trim_Galore v. 0.4.0 with removing reads that have length less than 35 bp after trimming. In addition, a base pair quality score cutoff (q=30) was used for filtering low quality base pairs. Reads were aligned against hg38 (UCSC version) using Bowtie2 v2.3.2. Secondary and supplementary alignments were removed, and only primary alignments were kept. Alignment reads were deduplicated to remove duplicate reads and keep unique reads using picard v. 1.9.1. Broad peaks were

identified from the alignment files using MACS2 v.2.1.1 with a cutoff score q<0.05.MACS2 called peaks at FRD 0.01 for individual samples and pooled samples were sub-jected to pathway analysis using the GREAT tool version 4.0.4 GO BP pathways enriched at FDR 0.05 were visualized as a network using Cytoscape 3.8.1 and EnrichmentMap 3.3.1 and Au-toAnnotate 1.3.3. The findMotifsGenome algorithm from HOMER v4.7 was used to identify known enriched motifs in genomic regions in each individual samples.

Flow Cytometry

Plots

Confirm that:

☒ The axis labels state the marker and fluorochrome used (e.g. CD4-FITC).

☒ The axis scales are clearly visible. Include numbers along axes only for bottom left plot of group (a 'group' is an analysis of identical markers).

☒ All plots are contour plots with outliers or pseudocolor plots.

☒ A numerical value for number of cells or percentage (with statistics) is provided.

Methodology

Sample preparation

Cells were dispensed in FACS buffer stained with specific antibodies incubated, washed and resuspended in FACS buffer

Instrument

BD Forttessa X-20, BD FACS Aria 3, Sony SH-800, Cell Sorter, MoFlo XDP Cell Sorter

Software

FlowJO_V10.7.1/ v7.7.1

Cell population abundance

	Starting cell count	Stem cell	Bulk cells
OCI_AML_8227	80-100 million	~5-10 million	~12-15 million
AML_patient samples	50-200 million	~ 500,000-1 million	~500,000-1 million
Cord blood samples CD34+	~5-6 million		
Cord blood samples CD34-	~40-60 million		

Gating strategy

The gating strategy was set as follows.
OCI_AML_8227 cells : FSC/SSC (represents the distribution of cells in the light scatter based on size and intracellular composition, respectively) to FSC Height/FSC width to SSC Height/SSC width (doublet discrimination) live gate (DAPI negative, viable cells within the sample analyzed) to CD34-APC-CY7/CD38-PE CY5 (identifies selective subsets, CD34+CD38- cells as Stem like and CD34-CD38+ as AML blasts).
AML Patient sample ROS High/Low: SC/SSC (represents the distribution of cells in the light scatter based on size and intracellular composition, respectively) to FSC Height/FSC width to SSC Height/SSC width (doublet discrimination) live gate (DAPI negative, the fraction of viable cells within the sample analyzed) to B PE-CD1/ PE-Cy7-CD3 to ex-clude the lymphocyte populations B515-CD45 (to identify the blast population) to CellROX deep red (ROS-low LSCs - the cells with the 20% lowest ROS levels and the ROS-high blasts - the cells with the highest 20% ROS levels).
Cord Blood :
HSPC (CD34+) SORT
HSC CD34+CD38-CD45RA-CD90+CD49f+
MPP CD34+CD38-CD45RA-CD90-CD49f-
MLP CD34+CD38-CD45RA+
CMP CD34+CD38+CD7+CD10-FLT3+CD45RA-CD71-
GMP CD34+CD38+CD7+CD10-FLT3+CD45RA+CD71-CD19-
MEP CD34+CD38-CD7-FLT3-CD45RA-CD71+CD19- (CD7 and CD10)

Mature cells SORT
B CD19+
T CD3+
NK CD19-CD3-CD56+
Gran CD19-CD3-CD14+CD15+
Mono CD19-CD3-CD14+CD15-

☒ Tick this box to confirm that a figure exemplifying the gating strategy is provided in the Supplementary Information.
