# Supplementary figures and images for: The metabolic enzyme hexokinase 2 localizes to the nucleus in AML and normal haematopoietic stem and progenitor cells to maintain stemness
Source: Nat Cell Biol. 2022 Jun 6;24(6):872–84. doi: 10.1038/s41556-022-00925-9 (PMC9203277; doi:10.1038/s41556-022-00925-9)

Figure 1A

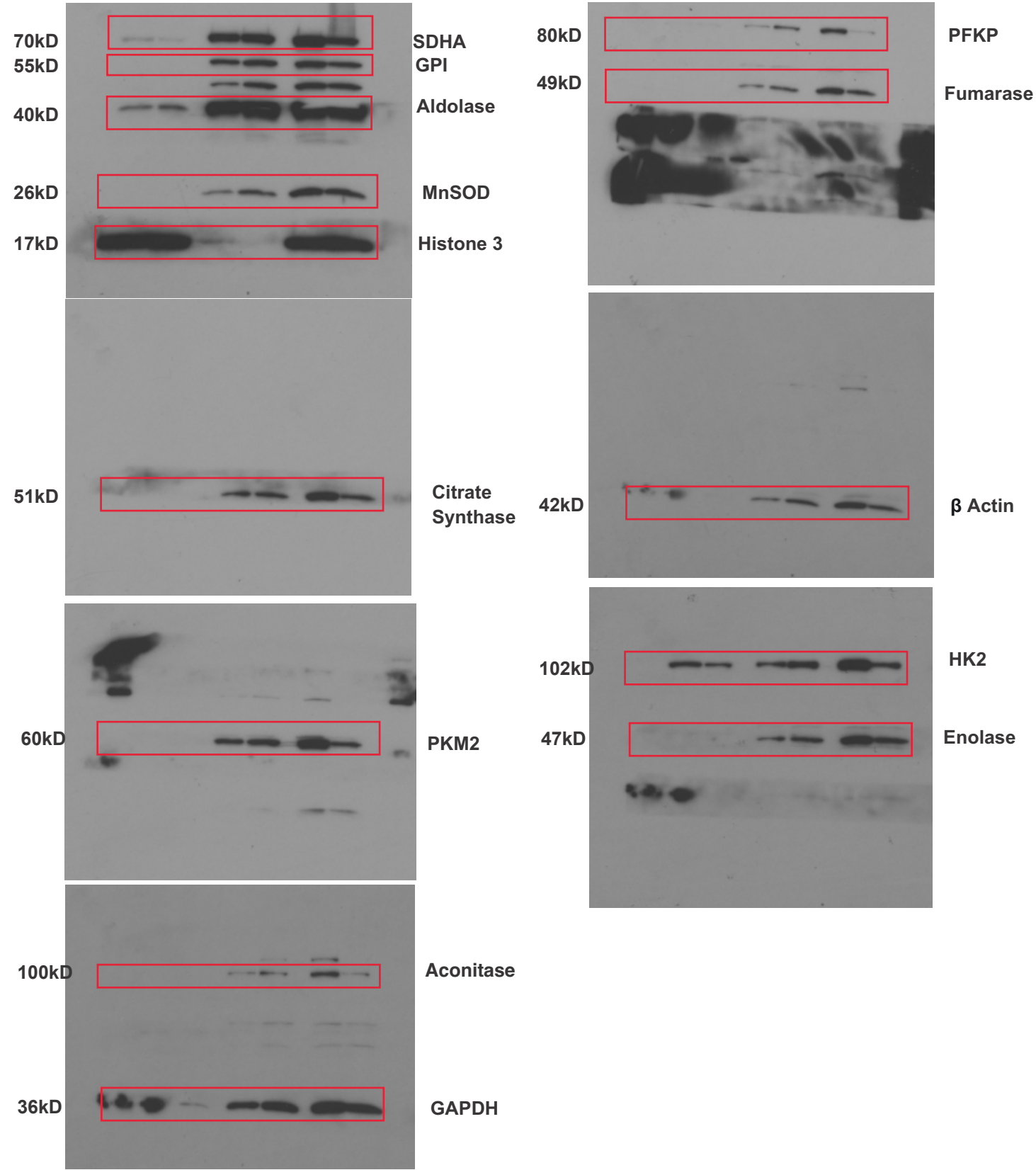

Supplement: Source Data Fig. 1 — Unprocessed western blots. [file 41556_2022_925_MOESM5_ESM.pdf]

Figure 4E

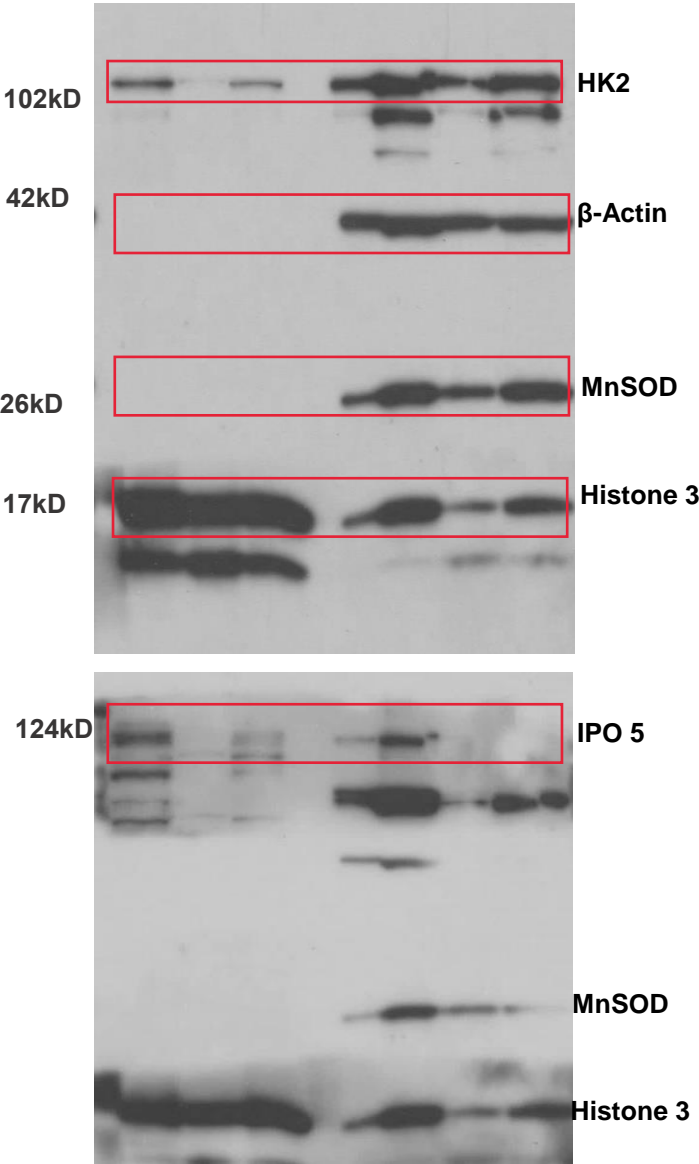

Supplement: Source Data Fig. 4. — Unprocessed western blots. [file 41556_2022_925_MOESM9_ESM.pdf]

Extended Data Figure 1B

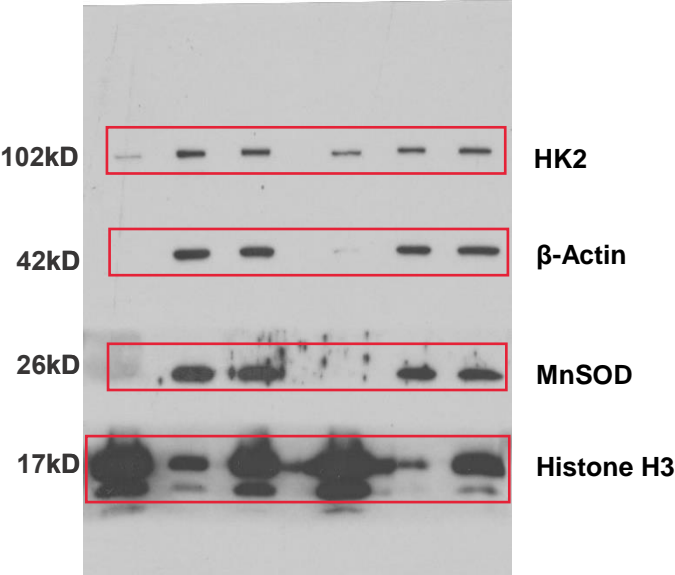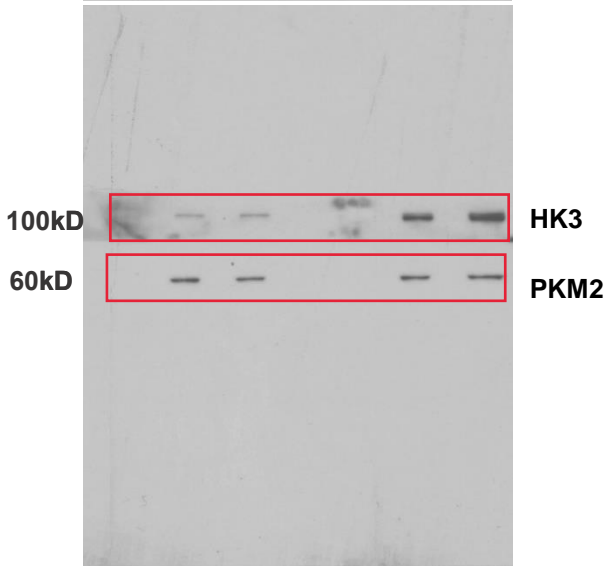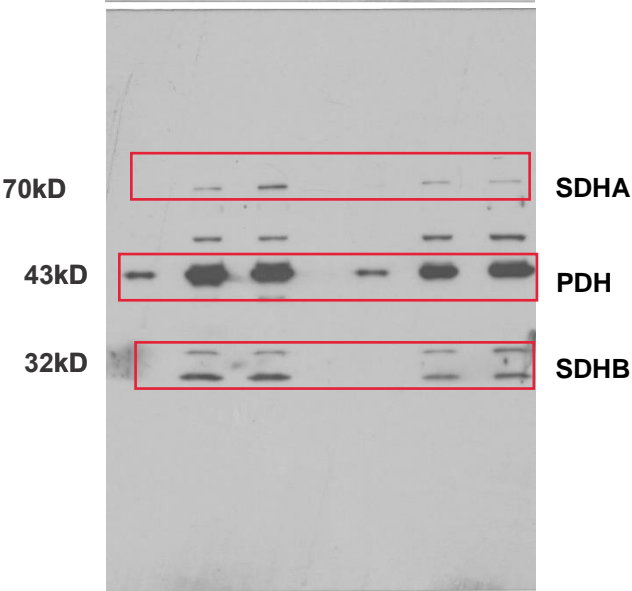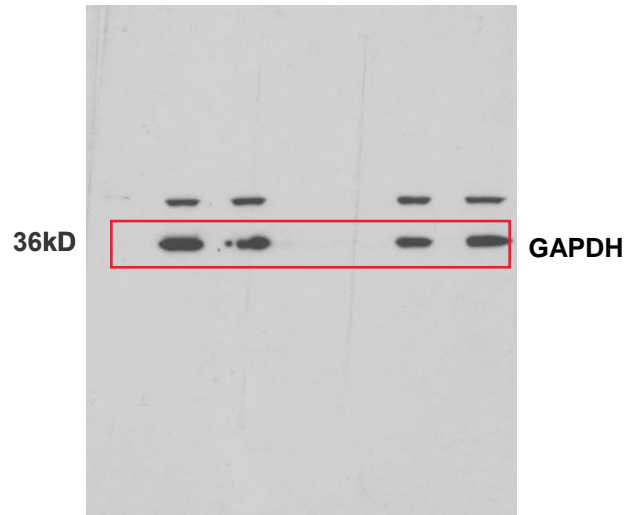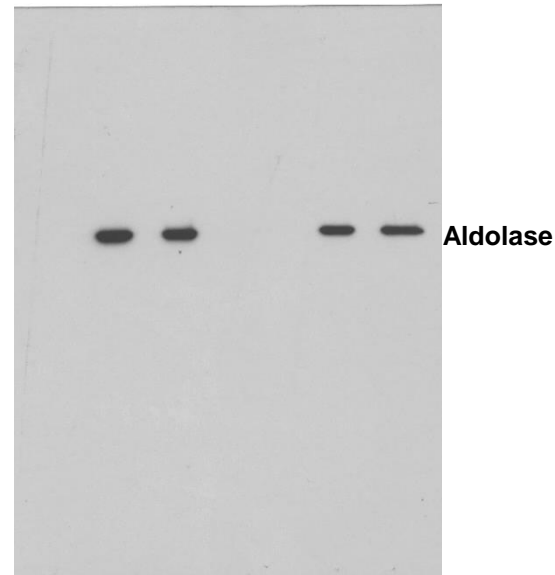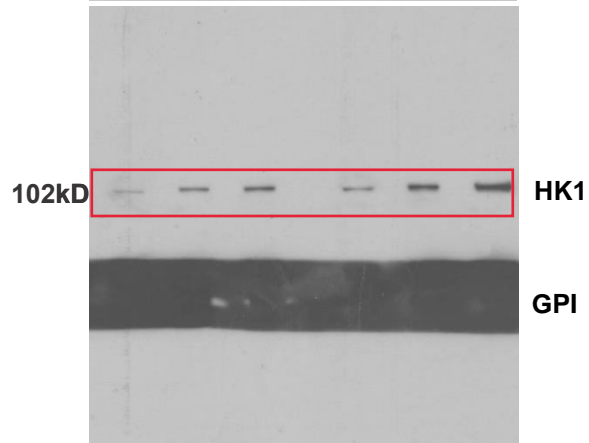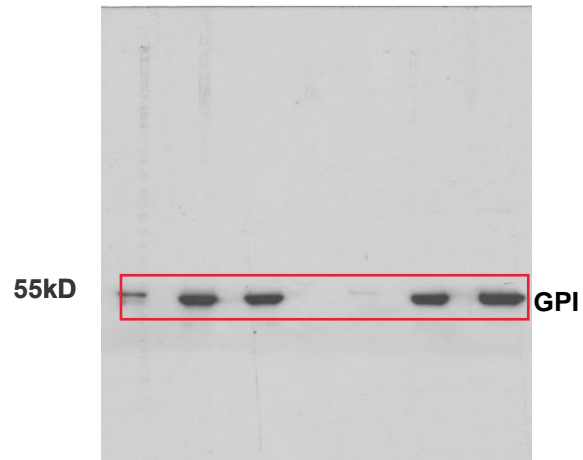

Extended Data Figure 1C

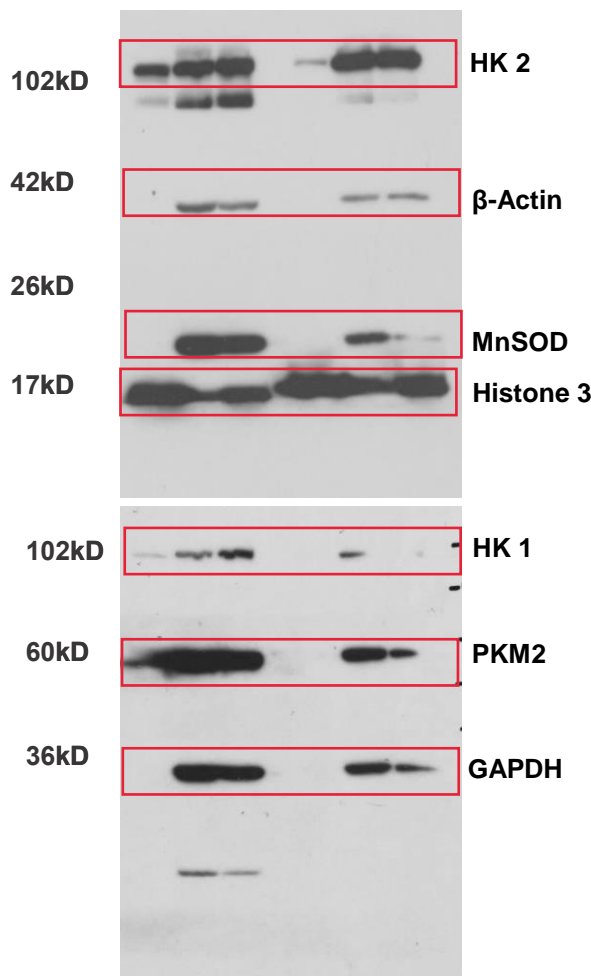

Extended Data Figure 1E

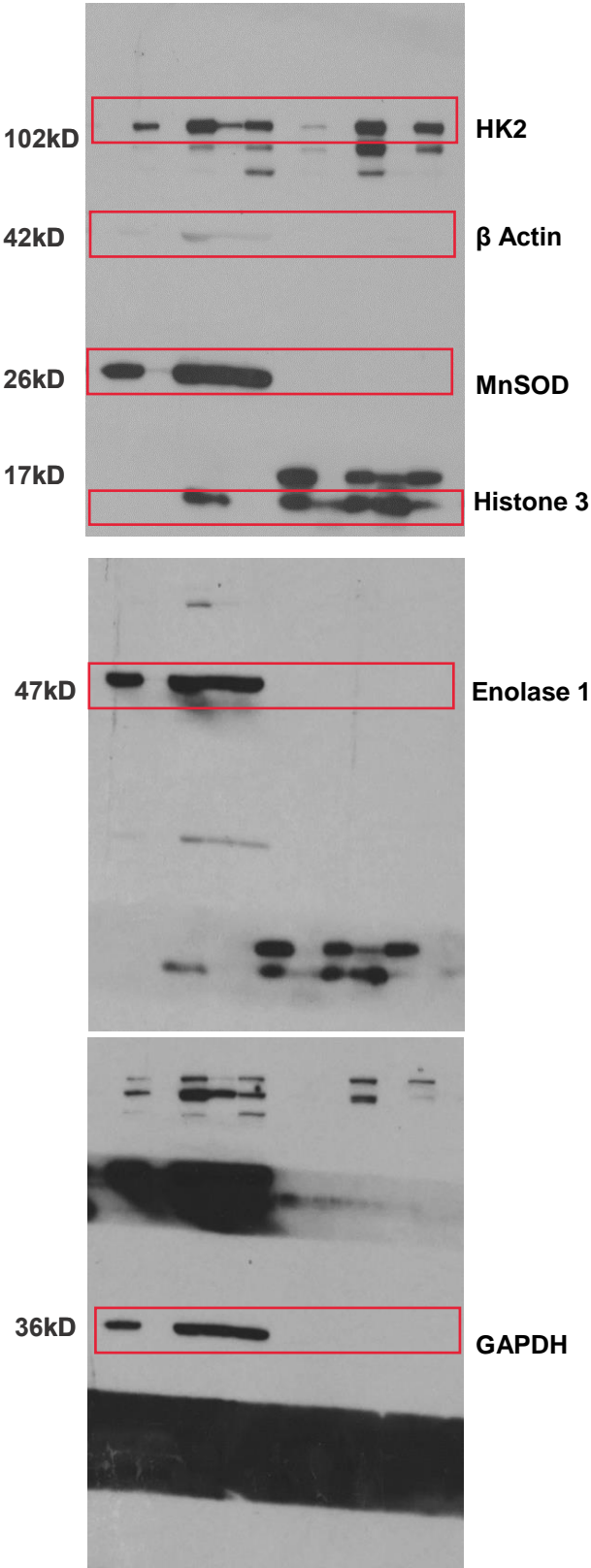

Extended Data Figure 1F

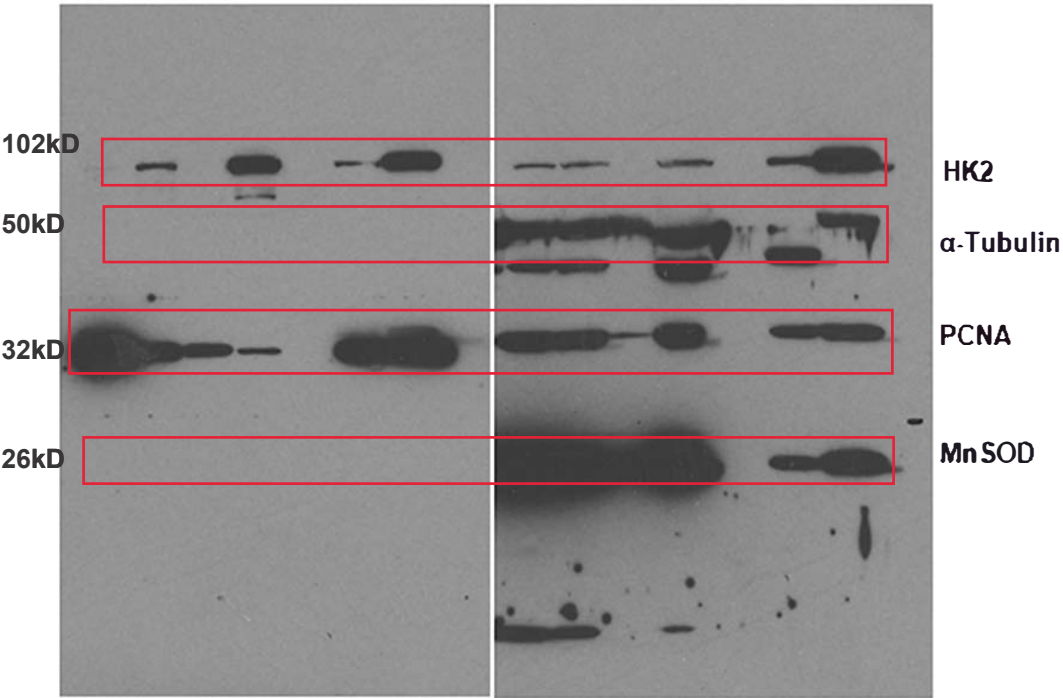

Supplement: Source Data Extended Data Fig. 1 — Unprocessed western blots. [file 41556_2022_925_MOESM13_ESM.pdf]

Extended Data Figure 2D

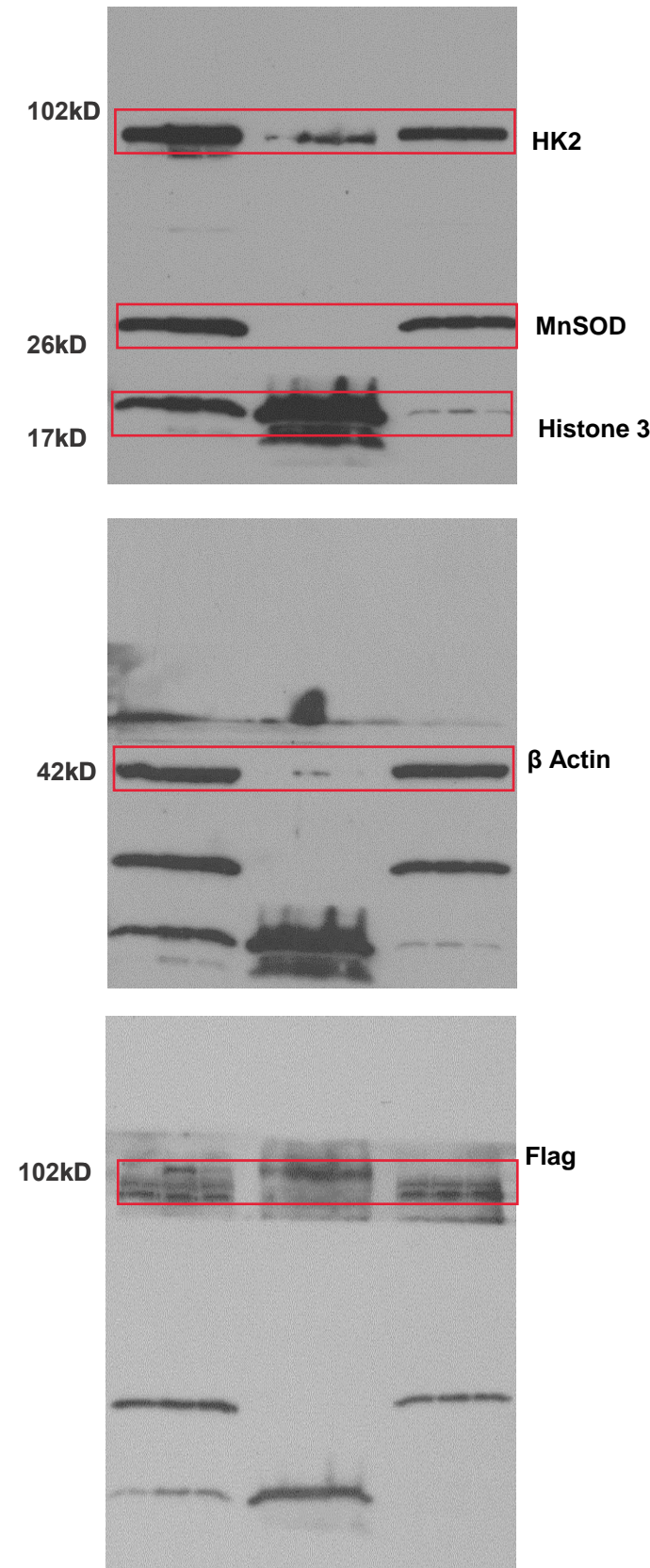

Extended Data Figure 2I

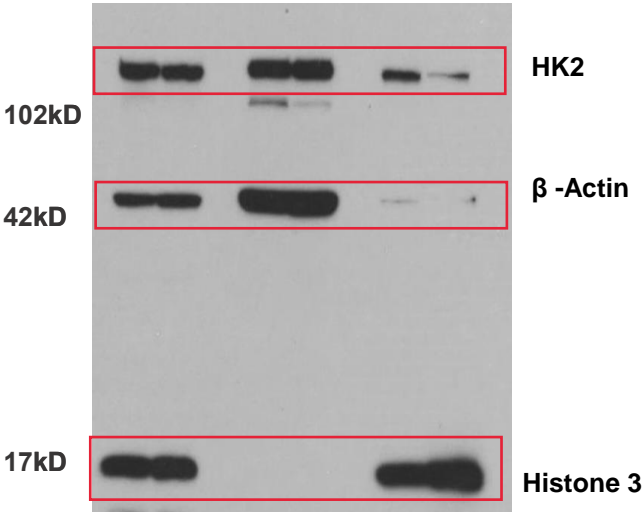

Extended Data Figure 2P

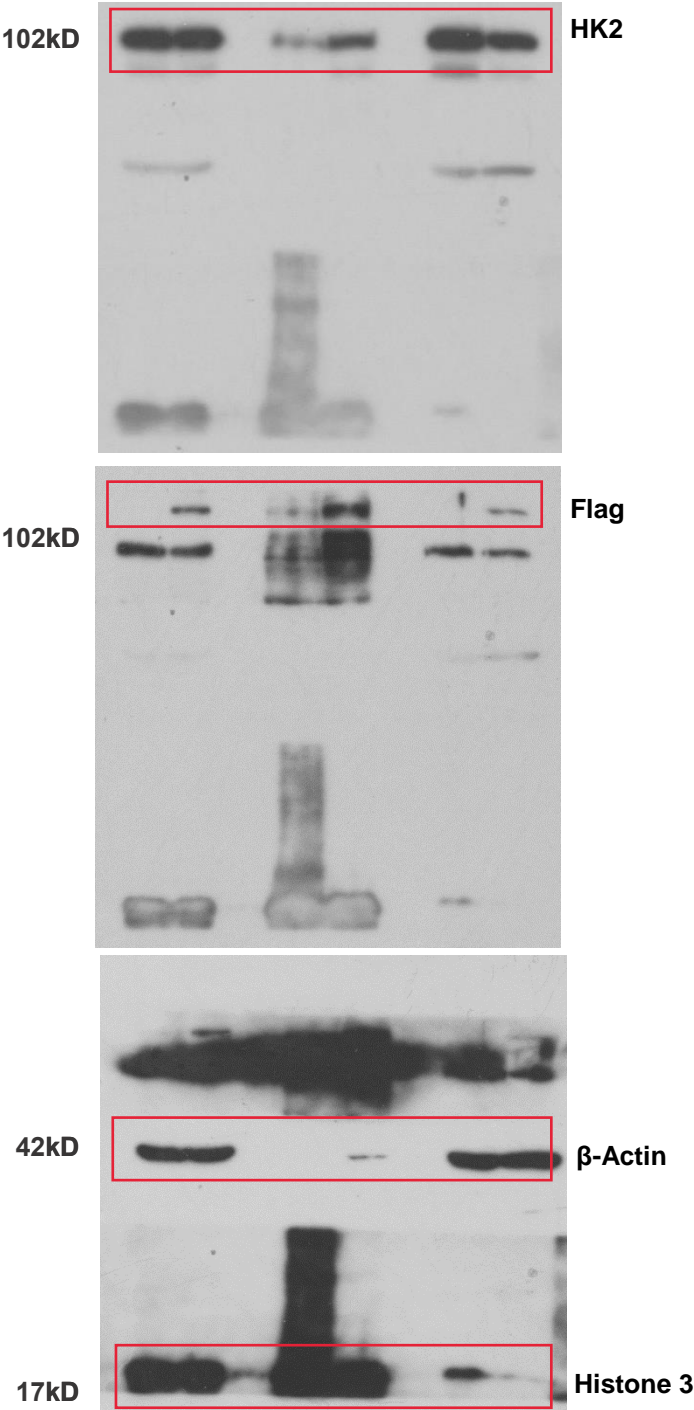

Supplement: Source Data Extended Data Fig. 2 — Unprocessed western blots. [file 41556_2022_925_MOESM15_ESM.pdf]

Extended Data Figure 3C

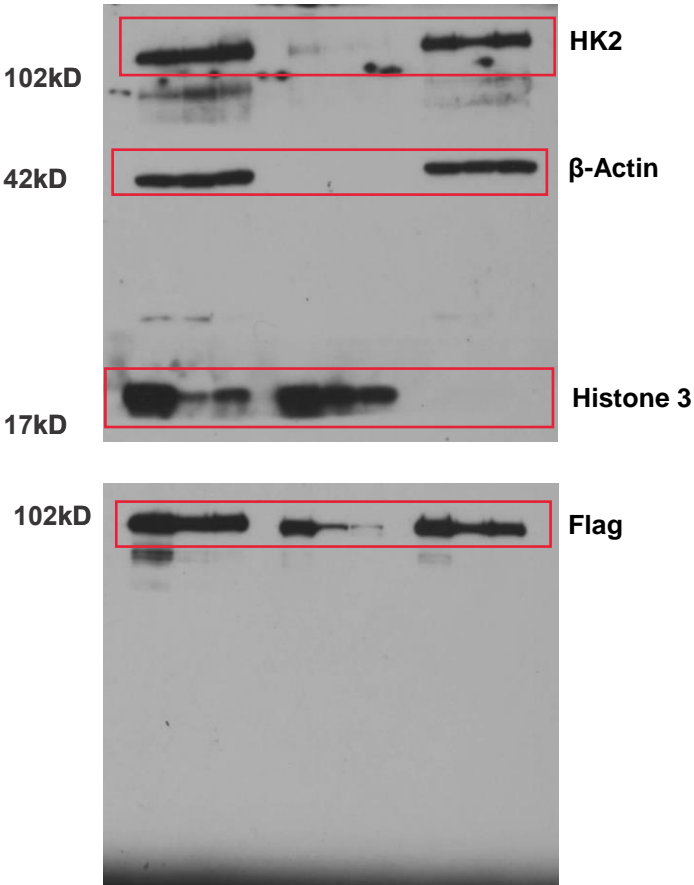

Extended Data Figure 3D

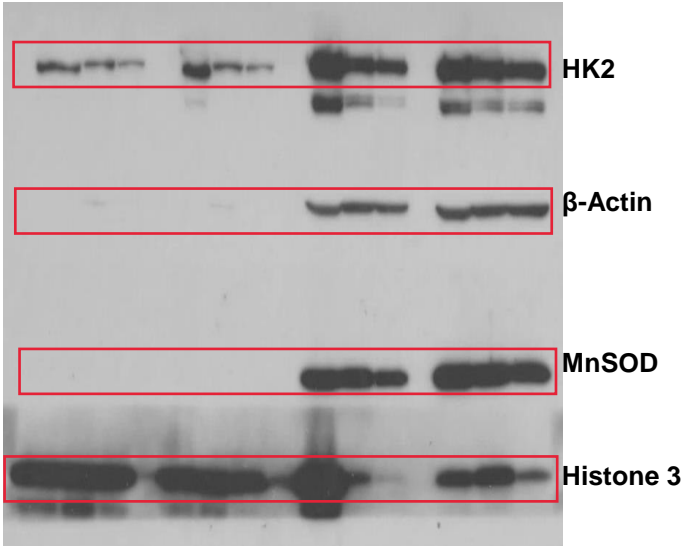

Extended Data Figure 3K

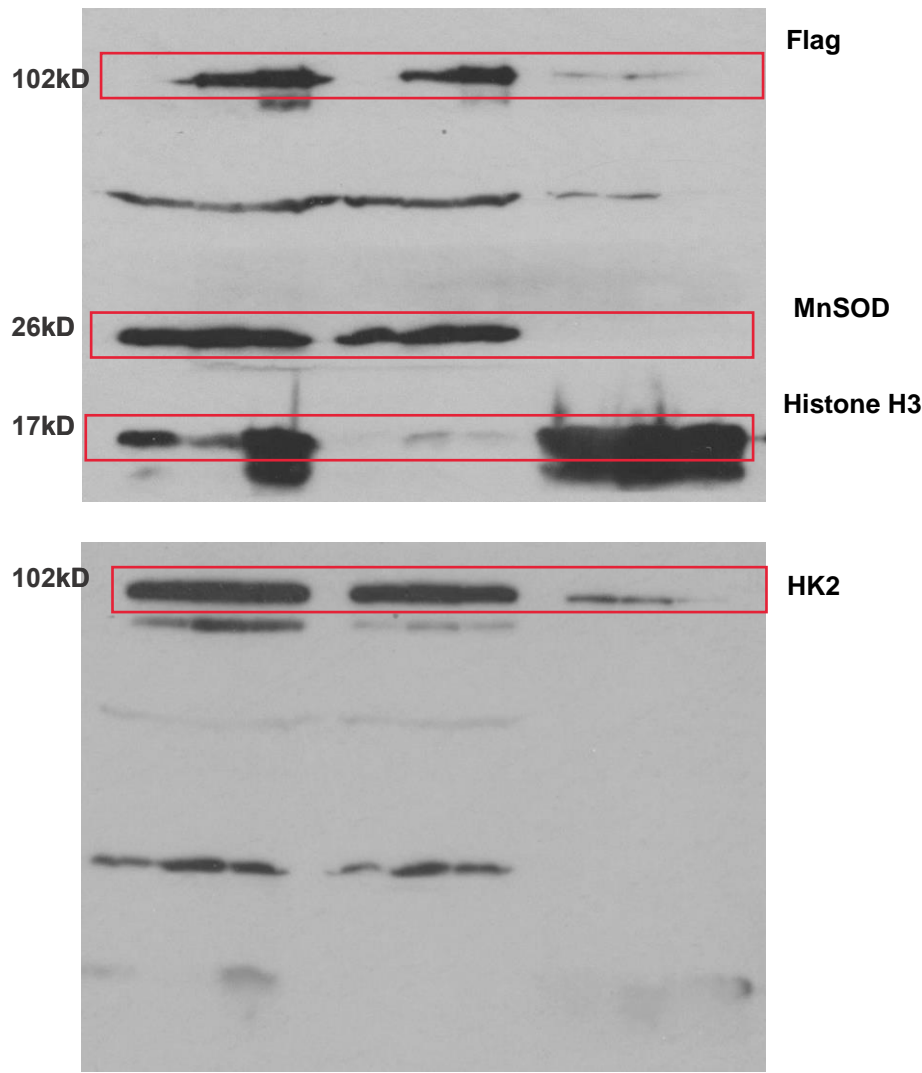

Supplement: Source Data Extended Data Fig. 3 — Unprocessed western blots. [file 41556_2022_925_MOESM17_ESM.pdf]

Extended Data Figure 5A

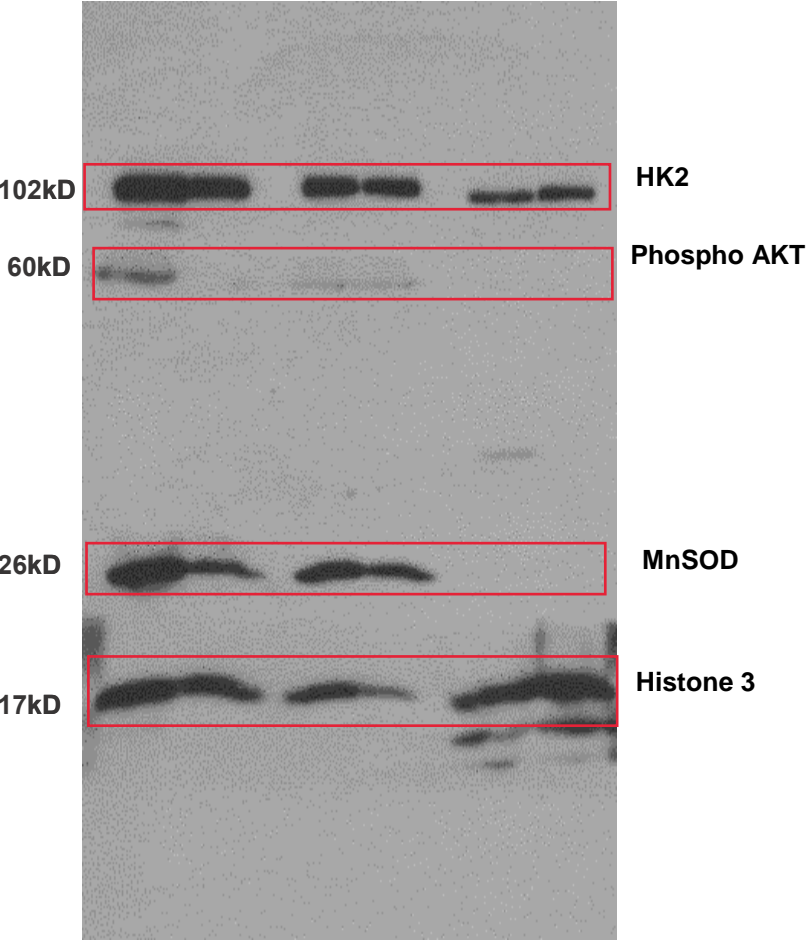

Extended Data Figure 5C

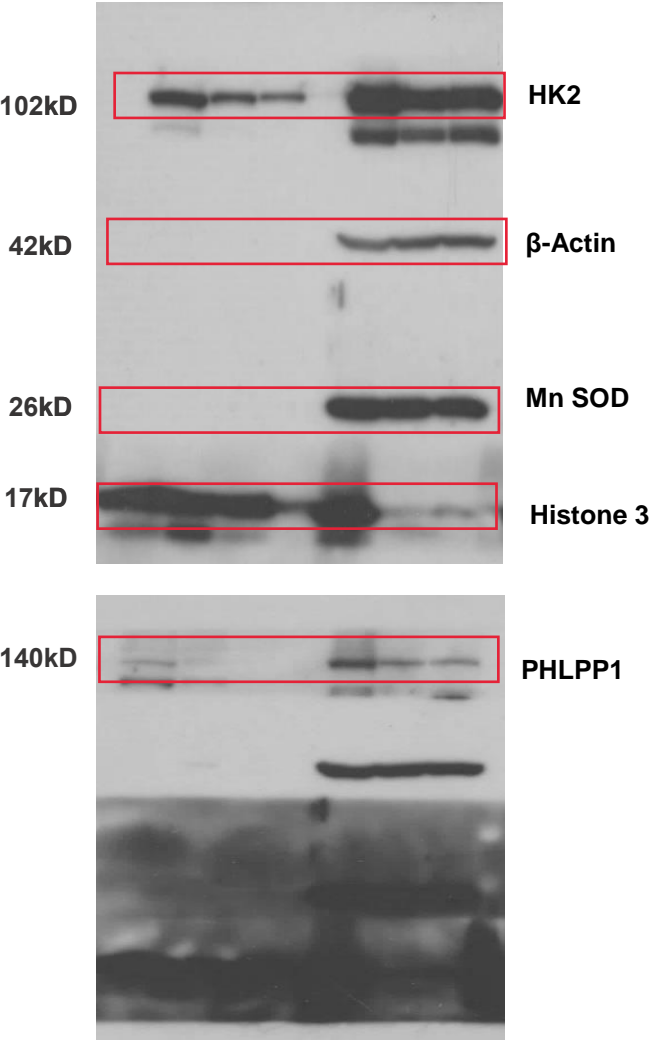

Extended Data Figure 5D

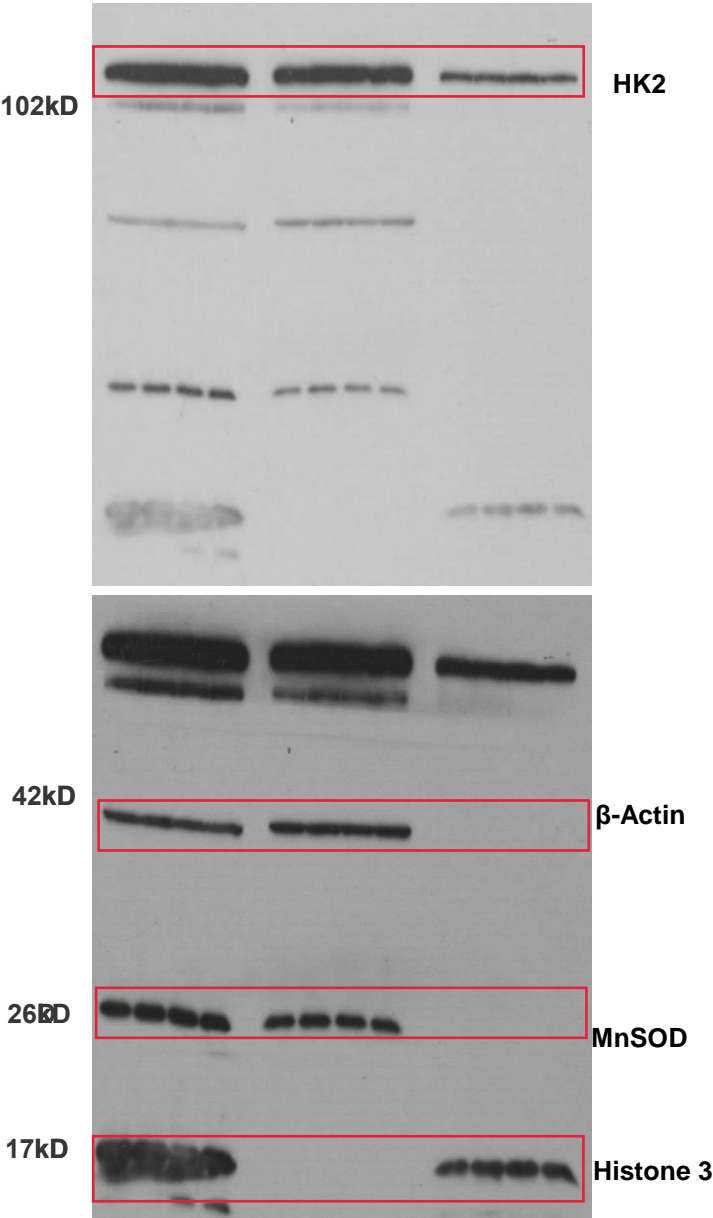

Extended Data Figure 5J

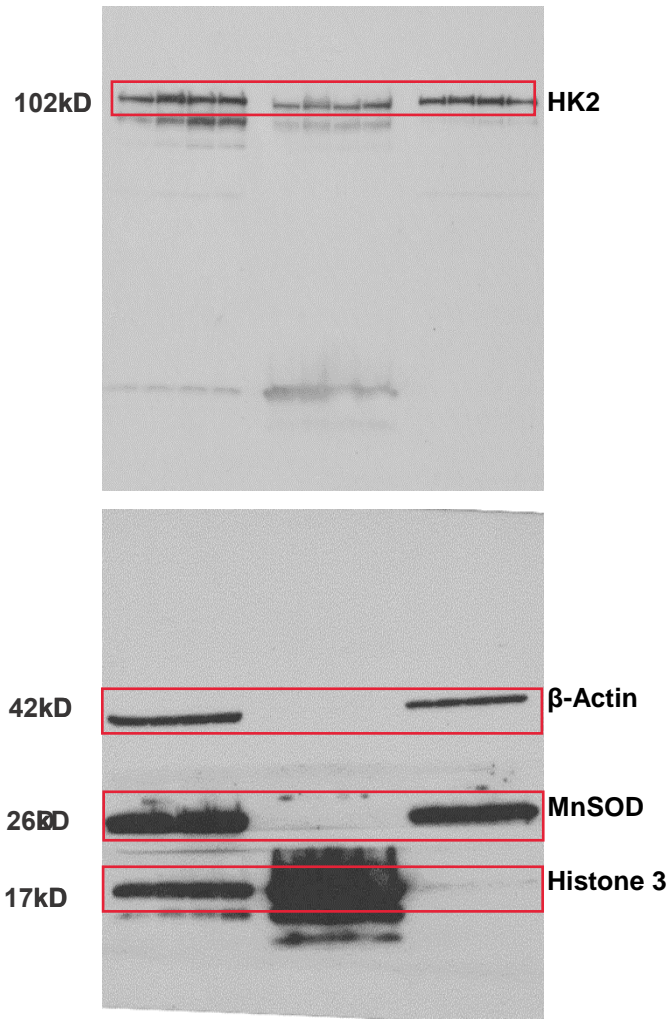

Supplement: Source Data Extended Data Fig. 5 — Unprocessed western blots. [file 41556_2022_925_MOESM20_ESM.pdf]
